# Later eating rhythm measured in children at 7 years of age in the ALSPAC cohort

**DOI:** 10.12688/wellcomeopenres.20605.2

**Published:** 2024-07-29

**Authors:** Mengxuan Zou, Laura Johnson, Sam Leary, Francisca Ibacache Fuentes, Kate Northstone

**Affiliations:** 1 University of Bristol Medical School, Bristol, England, BS8 2BN, UK; 2 Population Health Sciences, Bristol Medical School, University of Bristol, Bristol, England, BS8 2PS, UK; 3 Bristol Dental School, University of Bristol, Bristol, England, BS2 0PT, UK; 4 Bristol Medical School, University of Bristol, Bristol, England, BS8 2BN, UK

**Keywords:** ALSPAC, children, diet, later eating rhythm

## Abstract

Later eating rhythm (LER) refers to later timing, greater energy intake (EI), and higher frequency of eating occasions (meal/snack) in the evening. The significance of LER in child health is becoming increasingly recognised. However, the lack of consensus regarding definitions of LER make it challenging to fully comprehend its role. This data note describes LER variables derived in the Avon Longitudinal Study of Parents and Children (ALSPAC), an ongoing birth cohort which enrolled 14,541 pregnant women living in Avon, UK, with an expected date of delivery between April 1991 - December 1992. When children were 7 years, parents completed a structured 3-day food diary, recording all foods/drinks consumed over 3 days (preferably 1 weekend day and 2 weekdays). Data was available for 7,285 children (50.1% response rate). A subsample of 4,869 children had exact time of eating occasions added to the existing database, which only included broad indications of eating timing based on 2-7 hour long meal slots. 13 LER variables were derived for the entire week and weekdays/weekend days separately. These comprise: 1) eating around individual bedtime (number days); 2) eating around average bedtime (0number days); 3) time of evening main meal (hrs:mins); 4) time of last eating occasion (hrs:mins); 5) EI in the evening (percentage of total daily energy intake, %TDEI); 6) EI within 2hrs before bedtime (%TDEI); 7) EI for evening main meal (%TDEI); 8) EI for evening snacks (%TDEI); 9) Night eating1 (NE1): eating over 30% of total daily energy intake after 18:00 (number days); 10) NE2: eating over 25% of total daily energy intake within 2hrs before bedtime (number days); 11) eating frequency after 17:00 (number of eating occasions); 12) regularity of dinner (0, 1, 2, 3 days); 13) frequency of evening snacks (number days). We describe the derivation, prevalence and inter-corelations between LER variables.

## Introduction

Recent studies have emphasized that timing of food intake, known as chrono-nutrition, may play an important role in adiposity development by demonstrating changes in energy regulation through circadian-driven processes, such as transport of lipids, glucose, and dietary proteins in the intestine ^1– 7^. Trials in adults have shown that eating late at night results in less-efficient energy metabolism and changes in metabolic markers such as decreased whole-body fat oxidation and reduced glucose tolerance and insulin secretion, thereby increasing the risk of metabolic syndrome ^8– 10^. In addition emerging studies in animals have shown that a wide variety of metabolic markers are affected by later eating such as adipokines, glucocorticoids, and clock genes ^11, 12^. Experimental trials in rodents suggested that mice restricted to feeding during light cycle developed obesity ^13^, whereas mice restricted to feeding during dark cycle were protected from obesity ^14^. Psychologically, a bigger evening main meal has been suggested to cause higher hunger and a lower suppression of appetite compared with a bigger breakfast in weight management ^11^. Thus, the time of day of energy intake (EI), in particular later in the evening around bedtime, has been highlighted as a particular concern for the development of adiposity.

The concept of later eating rhythms has previously been studied in many different ways. Night Eating (NE) was first proposed by Stunkard *et al*. ^15^ in 1955, when they defined it as consumption of more than 25% of total daily energy intake (TDEI) after the evening meal. NE was commonly considered as the main criterion for diagnosing night eating syndrome (NES), a disordered eating behaviour, generally diagnosed as morning anorexia, evening hyperphagia, a depressed mood and insomnia in adults ^16^. Stunkard *et al*. modified these criteria in 1996 to “50% of TDEI or more after 19:00” ^17^. Later on, several researchers adapted this into a number of diverse definitions taking into account cultural differences, such as “the largest food intake occurring during a time period after 19:00” or “overeating following the evening meal but before the end of the sleep period”. However, these definitions were restricted to adults as NES does not typically appear in childhood, when eating times are largely determined by parents ^18, 19^. In addition, it is essential to differentiate NES/NE as psychiatric conditions from habitual later eating with and without overt medical problem. Therefore, other terms such as, “late-night snacking”, “eating late in the evening” ^20, 21^, “nocturnal eating” ^22^, “late-night overeating” ^23^, “evening chronotype” ^22^ and “night time eating/EI” ^24– 26^ have been used to capture one or more specific features of habitual later eating behaviours and distinguish them from NES. Definitions across studies are diverse, differing in the definition of “late”, and perspectives such as, timing of food intake, EI, and meal frequency in the evening/night. Therefore, to harmonize the inconsistent use of terms and to cover later eating behaviours comprehensively from all perspectives, a broader term “later eating rhythm” (LER) encompassing NE and eating more in the later part of the day was created to describe later eating behaviours in children and adolescents by our recent systematic review ^27, 28^.

This paper describes the LER variables that have been derived from parent-reported diet diaries over three days collected at age 7 years as part of the Avon Longitudinal Study of Parents and Children (ALSPAC). Given the growing evidence of the importance of LER for obesity-related eating behaviours and obesity in later life, these data can be used by studies focusing on chrono-nutrition, disordered eating, other time-related eating behaviours, and metabolic health in children.

## Methods

### Sample size

ALSPAC is an ongoing birth cohort study that enrolled 14,541 pregnant women living in Avon, UK, with an expected date of delivery between April 1991 and December 1992. The subsequent children (n = 14,062 at birth; n = 13,988 alive at 1 year) have been followed using questionnaires completed by parents and the children, educational records, and hands-on assessment at dedicated research clinics. An overall total of 913 additional children were recruited at age 7 years (n=456), age 8-18 years (n=262) and age 19-26 years (n=195) to bolster the initial sample, increasing the total child cohort to 14,901 alive at 1 year. By age 7 years, 546 children were permanently lost to follow-up, leaving 13,972 children eligible for follow-up at 7 years. A detailed account of the study methodology can be found elsewhere ^29, 30^. Ethical approval for the overall study was obtained from the ALSPAC Law and Ethics Committee (ALEC) and the local research ethics committees (IRB00003312). Ethical approval for the 7 year clinic was obtained from the United Bristol Healthcare Trust (E4168), Southmead (North Bristol Trust; Project 084/99) and Frenchay (North Bristol Trust; Project 99/42). Written informed consent for the use of data collected via questionnaires and clinics was obtained from participants following the recommendation of ALEC at the time.

**Figure 1.**
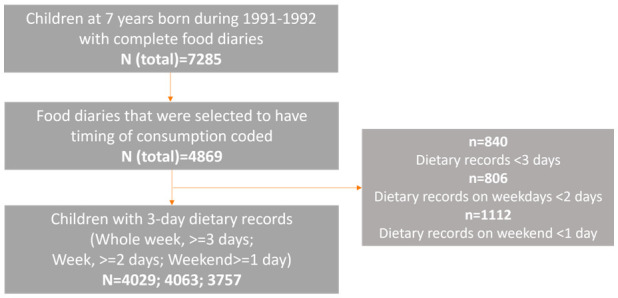
The sample size flow diagram for investigating later eating rhythm by the type of day.

3-day food diaries were collected as part of a face to face clinic at the age of 7 years ^31^. Parents were invited by post to complete a structured 3-day food diary and were asked to bring the completed food records to their clinic visit. Of all of those invited to the research clinic (n = 13,146), a total of 7,285 children had food diaries completed, resulting in a response rate of 55.4% ( Figure 1).

### Diet diary data

In each diet diary, parents were asked to record the exact time, name and size of any drinks, medicines and foods their children consumed over 3 individual days (preferably 1 weekend day and 2 weekdays), both in and out of the home. Parents were instructed to speak to teachers or carers regarding meals consumed away from home.

### Data preparation

***Time data entry*.** The exact time of consumption for every food or drink was not originally entered into the electronic dietary dataset, instead, general meal times were coded according to the rules described in Table 1. For example, when a food or drink was consumed at 8:15, the time of consumption was originally entered as “Breakfast, 0800”. However, the exact time of food intake is of particular importance for estimating LER variables and this was therefore keyed in at a later date.

**Table 1.** Time periods table original from ALSPAC coding documents.

Reported time of consumption in the raw data (diary)	Implicit meal-slot	“Original time” in dataset
Midnight to 6.59 a.m.	First thing	06:00
7.00 a.m. to 9.59 a.m.	Breakfast	08:00
10.00 a.m. to 11.59 a.m.	Mid-morning	11:00
12.00 noon to 2.29 p.m.	Lunch	13:00
2.30 p.m. to 4.59 p.m.	Tea	17:00
5.00 p.m. to 7.29 p.m.	Evening meal	20:00
7.30 p.m. to 11.59 p.m.	Late evening	22:00

The original paper diet diaries were retrieved from the archive for data entry. The process of time entry in the database was undertaken as follows:

1. The original diary ID number and day number were entered.

2. The foods listed for the first meal slot in the original data file were assessed to identify whether they matched with the foods reported in the original diet diary for the first exact time reported.

3. If all the foods reported matched, then the exact time for that eating occasion was entered next to each food. Time was entered as a four-digit number in 24-hour clock format.

4. A record was kept when any of the following occasions happened:

- If the person consumed the same food more than once in the same meal time slot and there was only one slot in the electronic diary, then the earliest eating occasion time was coded, and a note was kept about this case.
- If the food in the hard copy diary could not be matched to the food item in the database directly:
  ○ If there was no time of consumption of a food item in the hard copy diary but the food was found in the database, then ‘99’ was entered under the column of Time.
  ○ If the food in the database was not found in the diary (which may be due to the wrong input of the food name), the number ‘88’ was entered under the column of Time.

To date, food diaries for a subsample of 4,869 out of 7,285 children have been selected and coded with exact time of each food/drink ( Figure 1). Among these, 1,834 were selected based on the availability of other data, while 3,035 were selected randomly. Of these, 840 children with less than three days recorded were excluded from the analysis for weekday and weekend days combined. Thus, a total of 4,029 children were included in the analysis for the whole week, with 644 of those having data for three weekdays rather than a combination of week and weekend days. Those children who had at least two weekday food diaries available were included in the analysis for weekdays (N=4,063), and those who had at least one weekend food diary were included in the analysis for weekend days (N=3,757). There were 3,385 children who had food diaries recorded both for the week and weekend, hence they were included in the analysis for week vs weekend comparison.

The characteristics of children included and excluded in this study are presented in Table 2. It can be seen that there are no differences in those with precise timings compared to the remainder of the cohort with diet diaries according to child sex, BMI at 7 years of age and maternal age and education at birth (all p>0.10).

**Table 2.** Baseline Characteristics of children with precise timings compared to the remainder with diet diary data at 7 years of age.

Characteristic	Those with precise timings (n=4,869)	Those without precise timings (n=2,416)
**Sex at birth** Male (51.0%) Female (49.0%) X ^2^=2.21 (p=0.138)	52.2% (1177) 47.8 % (1079)	50.1% (1586) 49.9% (1578)
**Child BMI at 7 years** Mean (sd) t=0.01 (p=0.982)	16.20 (2.08)	16.27 (2.07)
**Maternal age (years)** Mean (sd) t=2.52 (p=0.112)	28.44 (4.61)	28.35 (4.51)
**Maternal education ^a^** < O level (20.8%) O level (35.7%) >= A level (43.5%) X ^2^=0.38 (p=0.828)	21.1% (823) 35.7 % (1397) 43.3% (1692)	20.5% (567) 35.7 % (989) 43.8% (1214)

^a^ O levels are compulsory exams taken at 16 years of age and A levels are optional exams taken at 18 years of age

### Defining LER in the ALSPAC cohort

***Eating occasion (meal/snack) definition*.** The definition of eating occasion (EO) will significantly influence the definition of LER variables. Therefore, the definitions of EO, meal and snack in the current study were developed based on our data in line with the approach used in previous work. As shown in Table 3, the current study defined EO as any occasion when food/drink was consumed, which was adopted by most previous studies with exact time of food intake in children ^32– 34^. Since food diaries do not usually identify participant-defined meals or snacks, previous studies tended to approximate breakfast, lunch, and dinner by applying “the biggest EO in the time slots that breakfast, lunch, and dinner usually fall into”, respectively ^32, 35, 36^.

**Table 3.** Definition of eating occasion (EO), meal and snack in the current ALSPAC study.

Definition applied	Description
**Eating occasion (EO)**	An individual EO is separated by the unique recorded time
**Main meals**	Largest EO (kcal) occurring between 6:00 and 11:59; 12:00 and 16:59; 17:00 and 23:59
**Snacks**	All other EOs other than the meals as defined above
**Morning snack; Afternoon ** **snack; evening snack**	Smaller EOs (kcal) occurring between 6:00 and 11:59; 12:00 and 16:59; 17:00 and 23:59 respectively
**Usual main meal times**	Breakfast: 6:00-9:59; lunch: 12:00-14:59; dinner: 17:00-18:29

**Figure 2.**
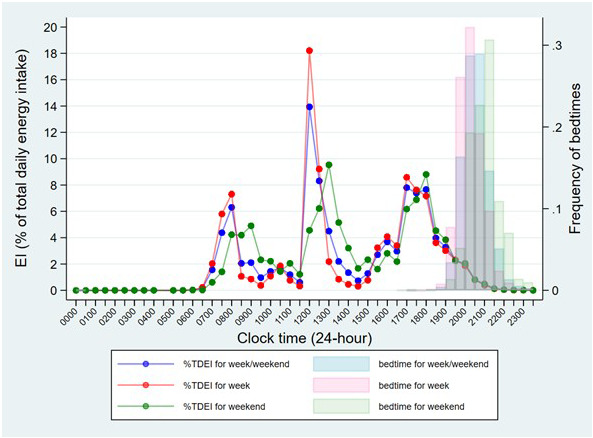
Summary of the mean energy intake for every 30-min time interval across the day and the distribution of bedtime by the type of day in children at age 7 years in ALSPAC. *Abbreviations: ALSPAC, Avon Longitudinal Study of Parents and Children; TDEI, total daily energy intake; EI, energy intake.

It is important to be able to assess whether the time slots we set for usual mealtimes was in line with when distinct large EOs tended to happen in our data. Thus, to gain insight into the usual timing of meals in this population, the mean EI (as a percentage of TDEI) was plotted for every 30-min time interval over a 24-hour period, across all participants ( Figure 2). Bedtime and type of day (week or weekend) were also taken into account. The resulting graph shows the lowest points of EI approaching 0, suggesting that suitable time cut-offs between morning, afternoon, and evening/night would be: 6:00-11:59, 12:00-16:59, and 17:00-23:59. Thus, main meals were determined as the biggest EOs in the morning, afternoon, and evening, similar to previous studies ^32, 35, 36^. All other smaller EOs were defined as snacks. In addition, the graph also displays three clear peaks in EI across the day, which suggests that the usual mealtimes for breakfast, lunch and dinner would be: 6:00-9:59, 12:00-14:59 and 17:00-18:29. Thus, main meal skipping was determined as “no EOs during the corresponding usual mealtimes”.

***Definitions and calculations for LER variables*.** Table 3 presents the various definitions of LER that were adopted in this study from three different perspectives: T (timing), E (energy intake), and F (meal frequency). Each domain includes different variables focusing on clock time, evening main meal, and evening snacks. The different definitions of EO (meal/snack) shown in Table 3 were applied. To minimize the impact of cultural differences, most LER variables were left in their original format without applying any categorical criteria (cut-offs).

As suggested by our review ^28^, the definition of “late” needs to be comparable when defining T1 (eating at a late time at night), or E1c (higher EI after a late time in the evening/night), and it is worth considering the time criteria relative to bedtime. In the current study, the parent-reported usual bedtime (hour:min) at around 7 years old (81 months) was used to indicate “late”. “Eating after the individual usual bedtime” and “EI within 2 hrs before bedtime” were therefore adopted. In addition, to maximise the sample size, “eating after the average bedtime” was used.

**Figure 3.**
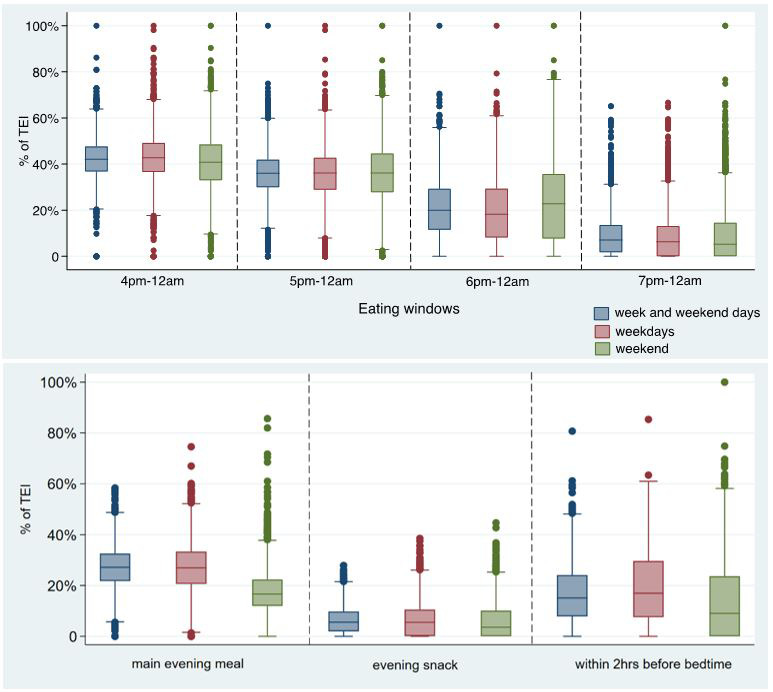
Percentage of TDEI for different meals/ time periods by weekdays or weekend days in the evening/night in children at age 7 years in ALSPAC. *Abbreviations: ALSPAC, Avon Longitudinal Study of Parents and Children; TDEI, total daily energy intake; EI, energy intake.

Notably, in the subgroup of E1 (EI after a specified time in the evening), time thresholds used in previous studies, such as 21:00 and 23:00, were so late that almost all UK children had no EI and had gone to bed ( Figure 2), hence they were not used in the current study. Others, such as EI after 16:00, 17:00, 18:00 and 19:00, were initially calculated. The practical meaning of these variables varied by country and was set depending on whether the researchers expected the evening main meal to be included ^20, 32, 37– 42^. Thus, the meaning of these clock-time related variables may be duplicated with other meal-type-related variables, and the clock time may mean different things for different countries. In order to reduce the duplication across variables and to increase comparability with previous research, box plots were used to detect the differences by exhibiting the quartiles of EI for different times (i.e., 16:00, 17:00, 18:00, 19:00, evening main meal, evening snacks, and 2 hours before bedtime). As shown in Figure 3, EI after 16:00 was similar to EI after 17:00, and the definition of evening in this study was 17:00–23:59, so the EI after 17:00 was kept referring to “EI in the whole evening”. Similarly, the distribution of EI for 19:00–23:59 and for evening snack were similar and EI for conventional meal/snack was used more commonly than the exact time of consumption, so EI for evening snack was kept.

Regarding the definition of NE, the time/energy criteria of NE varied between countries (e.g., consuming >25% or 50% of TDEI after 19:00, 20:00 or 21:00). Despite these variations, studies consistently reported a prevalence of NE ranging from 12.8% to 37.0% ^32, 39– 42^. Thus, an appropriate definition of NE aligns with the eating culture of the target population. In the current study, very few children consumed 50% of TDEI in the evening regardless of the time criteria, so 50% of TDEI was excluded as energy criteria. Additionally, 17:00–23:59 was too broad, and 19:00 and later was too restrictive to be considered for the time criteria of NE, as most children consumed over 25% of TDEI after 17:00, and almost all children had less than 25% of TDEI after 19:00. Thus 18:00 and “2 hours before bedtime” were kept as time criteria. Taking the reference prevalence (12.5%–37%) into account, the 75th percentile of TDEI was used to determine the final exact energy criteria, thus 30% of TDEI for 18:00–23:59 and 25% of TDEI within 2 hours before bedtime were finally adopted. Hence the definitions of NE for the UK children in ALSPAC were adopted as: E1c1 (NE1), the frequency of consuming over 30% of TDEI after 6pm (E1c1), and E1c2 (NE2), frequency of consuming over 25% of TDEI within 2 hours before bedtime. Therefore, EI between 16:00 and 23:59, 18:00 and 23:59, 19:00 and 23:59 and 21:00 and 23:59 were excluded.

Consequently, a total of 13 LER variables (T1-a/b, T2, T3; E1-a/b/c1/c2, E2, E3; F1, F2, F3) were considered in the current study. The variables were calculated for the whole week, weekdays only, and weekend days only as follows:

•    Timing: All the recorded times for food intake from the 24-hour clock were transformed to minutes which enabled the calculation of the mean values. The mean timing for the EO was then calculated by dividing the total timing for the EO over n days by the number of recorded days (n). “n” could be the overall number of weekdays (n≥2), weekend days (n≥1), or weekdays and weekend days combined (n=3), depending on the type of day. “k” was the specific recorded day (k=1, the first day; k=2, the second day; k=3, the third day). For easier interpretation of the results, the units needed to be transformed from minutes to hours, where appropriate.

$$Timing\;of\;an\;EO\;(mins)\;=\;\frac{\sum_{k=1}^{n}Timing_{Day\_\text{k}}\;(mins)}{n}$$

•    EI: The mean EI from the EO of interest as a percentage of total daily energy intake was calculated by dividing the overall energy from the EO over n days by the total energy intake over n days. “n” could be the number of weekdays (n≥2), weekend days (n≥1), or weekdays and weekend days combined (n=3), depending on the type of day.

$$Daily\;Energy\;Intake\text{ (}kcal\text{)}\;=\;\frac{\sum_{k=1}^{n}TDEI_{Day\_\text{k}}\;(kcal)}{n}$$

$$Mean\;EI\;from\;an\;EO\;or\;time\;frame\text{ (\%TDEI)}\;=\;\frac{\sum_{k=1}^{n}EI\;from\;a\;meal\;or\;time\;frame_{Day\_\text{k}}\;(kcal)}{\sum_{k=1}^{n}TDEI_{Day\_\text{k}}\;(kcal)}\;\;\times \;\;100\%$$

•    NE: The frequency of being a night eater was calculated as the number of recorded days an individual met the criteria of NE. For weekdays and weekend days combined, four categories were created: 0 days, 1 day, 2 days, and 3 days. For weekdays, three categories were created: 0 days, 1 day, ≥ 2 days. For weekend days, two categories were created: 0 days, ≥ 1 day.

•    MF: (1) The frequency of EOs, whether for the whole day or for the evening/night, as count variables, was calculated as the median number of EOs over the recorded days. (2) The frequency of evening main meals and evening snacking were calculated as the number of recorded days consuming an evening main meal and evening snacking, respectively. Four categories were created for week/weekend: 0 days, 1 day, 2 days, and 3 days; three categories for weekdays: 0 days, 1 day, ≥ 2 days; and two categories for weekend days: 0 days, ≥ 1 day.

***Statistical analysis*.** LER variables were generated using Stata v.17.0. Histograms were used to examine the distribution of continuous variables. Continuous variables (T2, T3, E1-a/b, E2, E3) were summarised using means and standard deviations (SDs) if they were normally distributed. The count variables (F1) and non-normally distributed continuous variables were summarised using medians and interquartile ranges (IQR 25th, 75th percentile). Ordinal/binary variables (T1-a/b, E1-c1/c2, F2, F3) were reported as percentages (%). The prevalence of LER was reported by the type of day (weekdays, weekend days, and the whole week). Paired t-tests for continuous variables, Wilcoxon matched-pairs signed-rank tests for count variables, and Pearson’s chi-square tests for categorical variables were used to estimate any differences between the week and the weekend. Spearman’s correlations were calculated to explore inter-correlations between the 13 LER variables.

### Key results

A total of 4,029 children (males 46%, females 54%) aged around 7.5 years (mean 90.4 SD 4.1 months) were included in this study. The average bedtime was 20:24, (20:00 in the week and 20:42 at the weekend). The median daily number of EOs was 6.0, which was similar to the number of conventional meals a day (breakfast, morning snack, lunch, afternoon snack, dinner, evening snack).

**Figure 4.**
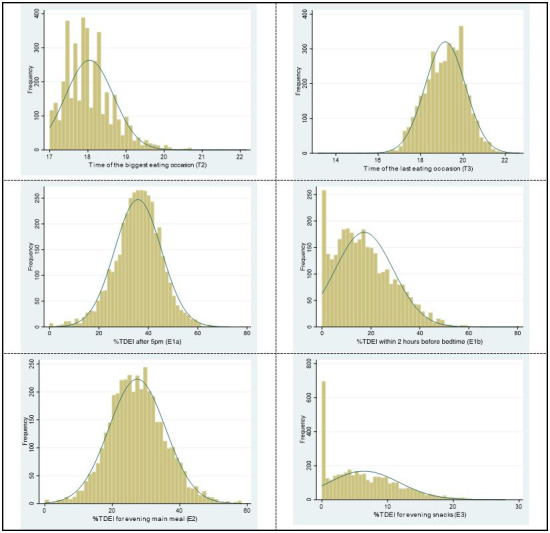
The distribution of continuous LER variables for week/weekend in ALSPAC. *Abbreviations: ALSPAC, Avon Longitudinal Study of Parents and Children; LER, Later eating rhythm.

The distribution of continuous LER variables is shown in Figure 4, three of them are skewed (T2, time of the biggest EO; E1b, EI within 2 hours before bedtime; E3, EI for evening snacks). Thus, they were reported as medians (IQR).

Table 5 describes the prevalence of LER by sex, and type of day. From the perspective of timing, more than half of children did not eat around bedtime on any of the days recorded, regardless of which bedtime (individual/average) was used. Children were more likely to eat before going to bed on weekdays than on weekends, (38.4% and 11.4%, respectively). In addition, the time of the greatest EO in the evening was around 18:00 (6 mins later on weekends). The last EO happened at 19:12 on average (3 mins later on weekends). Sex differences were observed in timing of the last EO only, with around 6 mins later in males than females. No substantial sex differences were found in other time-related LER variables.

**Table 5.** Description of LER by the type of day according to timing, energy intake and meal frequency in ALSPAC.

LER variables ^†^	Week/Weekend (3 days)					Week (>=1 day)					Weekend (>=1 day)					Difference between week and weekend (Total) ^b^
	Male n=1849	Female n=2170	Total n=4019	Difference in means/proportions (95%CI)	*p*-value ^a^	Male n=1570	Female n=1815	Total n=3385	Difference in means (95%CI)	*p*-value ^a^	Male n=1570	Female n=1815	Total n=3385	Difference in means (95%CI)	*p*-value ^a^	
**Timing**																
**T1a:** Eating around individual bedtime ^*^, n (%)																
0	865 (57.3)	1018 (56.7)	1883 (57.0)	-	0.943	784 (60.8)	933 (62.0)	1717 (61.4)	-	0.541	1147 (88.9)	1325 (88.4)	2472 (88.6)	-	0.707	<0.001 ^b1^
1 day	349 (23.1)	422 (23.5)	771 (23.3)			505 (39.2)	573 (38.1)	1078 (38.6)			144 (11.2)	174 (11.6)	318 (11.4)			
>= 2 days ^c^	295 (19.6)	354 (19.7)	649 (19.7)													
**T1b:** Eating around average bedtime, n (%)																
0	1279 (69.2)	1543 (71.1)	2822 (70.2)	-	0.391	1155 (73.6)	1386 (76.4)	2541 (75.1)	-	0.061	1428 (91.0)	1632 (89.9)	3060 (90.4)	-	0.307	<0.001 ^b1^
1 day	357 (19.3)	398 (18.3)	755 (18.8)			415 (26.4)	429 (23.6)	844 (24.9)			142 (9.0)	183 (10.1)	325 (9.6)			
>= 2 days ^c^	213 (11.5)	229 (10.6)	442 (11.0)													
**T2:** Time of the biggest EO (hr), median (IQR)	18.0 (17.6, 18.4)	18.0 (17.6, 18.3)	18.0 (17.6, 18.4)	-	0.753	17.9 (17.5, 18.4)	17.9 (17.5, 18.4)	17.9 (17.5, 18.4)	-	0.667	18.0 (17.5, 18.5)	18 (17.5, 18.5)	18.0 (17.5, 18.5)	-	0.172	<0.001 ^b2^
**T3:** Time of the last EO (hr), mean (SD)	19.2 (1.0)	19.1 (0.9)	19.2 (1.0)	0.1 (0.0, 0.2)	0.001	19.2 (1.1)	19.1 (1.1)	19.1 (1.1)	0.1 (0.0, 0.2)	0.009	19.2 (1.2)	19.2 (1.2)	19.2 (1.2)	0.05 (-0.0, 0.1)	0.191	0.002 ^b3^
**EI (%TDEI), mean (SD)**																
**E1a:** EI after 17:00	36.2 (9.2)	35.5 (9.7)	35.8 (9.5)	0.6 (0.1, 1.2)	0.035	35.8 (11.5)	35.3 (11.8)	35.5 (11.7)	0.5 (-0.3, 1.3)	0.234	36.3 (13.0)	35.9 (14.1)	36.1 (13.6)	0.4 (-0.5, 1.4)	0.349	0.038 ^b3^
**E1b:** EI within 2hrs before bedtime ^*^, median (IQR)	15.8 (8.4, 25.0)	15.5 (7.7, 25.1)	15.6 (8.1, 25.1)	-	0.499	17.0 (7.9, 29.4)	16.9 (7.2, 30.0)	16.9 (7.5, 29.6)	-	0.671	9.4 (0.0, 22.8)	8.6 (0, 24.6)	9.0 (0, 23.6)	-	0.593	<0.001 ^b2^
**E2:** EI for evening main meal	27.3 (8.3)	27.5 (8.5)	27.4 (8,4)	-0.2 (-0.7, 0.3)	0.407	26.7 (9.9)	27.3 (10.1)	27.0 (10.0)	-0.5 (-1.2, 0.2)	0.138	17.5 (8.3)	17.9 (9.8)	17.7 (9.1)	-0.5 (-1.1, 0.1)	0.127	<0.001 ^b3^
**E3:** EI for evening snack, median (IQR)	6.1 (2.4, 10.4)	5.2 (1.5, 9.5)	5.5 (1.9, 9.8)	-	<0.001	6.0 (1.0, 11.2)	5.2 (0.0, 10.0)	5.5 (0.1, 10.5)	-	<0.001	4.2 (0, 10.7)	3.1 (0, 9.7)	3.6 (0, 10.1)	-	0.017	<0.001 ^b2^
**Night eating), n (%)**																
**E1c1:** >=30% of TDEI after 18:00																
0	714 (38.6)	855 (39.4)	1569 (39.0)	-	0.448	887 (56.5)	1087 (59.9)	1974 (58.3)	-	0.046	969 (61.7)	1080 (59.5)	2049 (60.5)	-	0.188	<0.001 ^b1^
1 day	642 (34.7)	755 (34.8)	1397 (34.8)			683 (43.5)	728 (40.1)	1411 (41.7)			601 (38.3)	735 (40.5)	1336 (39.5)			
2 days	358 (19.4)	429 (19.8)	787 (19.6)													
3 days	135 (7.3)	131 (6.0)	266 (6.6)			-	-	-			-	-	-			
**E1c2:** >=25% of TDEI within 2hrs before bedtime ^*^																
0	635 (42.4)	730 (41.2)	1365 (41.8)	-	0.885	664 (51.5)	769 (51.1)	1433 (51.3)	-	0.812	981 (76.0)	1111 (74.1)	2092 (75.0)	-	0.255	<0.001 ^b1^
1 day	436 (29.1)	514 (29.0)	950 (29.1)			625 (48.5)	737 (48.9)	1362 (48.7)			310 (24.0)	388 (25.9)	698 (25.0)			
2 days	294 (19.6)	364 (20.6)	658 (20.1)													
3 days	134 (8.9)	162 (9.2)	296 (9.1)			-	-	-			-	-	-			
**Meal frequency, n (%)**																
**F1:**Counts of EOs after 17:00, median (IQR)	2.0 (2.0, 3.0)	2.0 (2.0, 3.0)	2.0 (2.0, 3.0)	-	<0.001	2.0 (1.5, 3.0)	2.0 (1.5, 3.0)	2.0 (1.5, 3.0)		<0.001	2.0 (1.5, 3.0)	2.0 (1.0, 3.0)	2.0 (1.0, 3.0)		0.041	0.165 ^b2^
**F2:** Frequency of dinner																
0	26 (1.4)	48 (2.2)	74 (1.8)	-	0.105	96 (6.1)	121 (6.7)	217 (6.4)	-	0.513	272 (17.3)	337 (18.6)	609 (18.0)	-	0.348	0.001 ^b1^
1 day	164 (8.9)	207 (9.5)	371 (9.2)			1474 (93.9)	1694 (93.3)	3168 (93.6)			1298 (82.7)	1478 (81.4)	2776 (82.0)			
2 days	523 (28.3)	645 (29.7)	1168 (29.1)													
3 days	1136 (61.4)	1270 (58.5)	2406 (59.9)			-	-	-			-	-	-			
**F3:** Frequency of evening snacks																
0	202 (10.9)	304 (14.0)	506 (12.6)	-	0.006	302 (19.2)	423 (23.3)	725 (21.4)	-	0.004	555 (35.4)	699 (38.5)	1254 (37.1)	-	0.058	P<0.001 ^b1^
1 day	379 (20.5)	487 (22.4)	866 (21.6)			1268 (80.8)	1392 (76.7)	2660 (78.6)			1015 (64.7)	1116 (61.5)	2131 (63.0)			
2 days	598 (32.3)	668 (30.8)	1266 (31.5)													
3 days	670 (36.2)	710 (32.7)	1380 (34.3)			-	-	-			-	-	-			

Abbreviations: EO, eating occasion; TDEI, total daily energy intake; EI, energy intake; IQR, interquartile range (25 ^th^, 75 ^th^ percentile). a: Mann-Whitney U test, unpaired t-test or Pearson’s chi-square test. b: b1, Pearson’s chi-square test; b2, Wilcoxon matched-pairs signed-rank test; and b3, paired t-test. c: very few children (<4%) ate around bedtime every day (3 days), thus children eating around bedtime for 2 days and 3 days were grouped together. * n=3303 on week/weekend days; n=2795 on weekdays; n=2790 on weekend days due to missing data in bedtime † Detailed definitions of LER variables can be found in Table 4.

**Table 4.** Definitions of potential later eating rhythm variables in ALSPAC.

Later eating rhythm	Variables in this study	Variable type	Definition (calculation)
Timing of EO	T1a: Eating around individual bedtime	Categorical data	Number of recorded days consuming any EO after usual individual bedtime (0-3 days for week/weekend; 0-2 days for weekdays; 0-1 days for weekend days)
	T1b: Eating around average bedtime	Categorical data	Number of recorded days consuming any EO after average bedtime, 20:03 on weekdays and 20:42 on weekend days (0-3 days for week/weekend; 0-2 days for weekdays; 0-1 days for weekend days)
	T2: Evening main meal	Continuous data	Time of the biggest eating occasion between 17:00-23:59 (hrs:mins).
	T3: Last eating occasion	Continuous data	Time of the last eating occasion of the day (hrs:mins).
EI of EO-continuous variables	E1a: %TDEI in the evening	Continuous data	Mean EI between 17:00-23:59 over the recorded days (%TDEI).
	E1b: %TDEI within 2hrs before bedtime	Continuous data	Mean EI within 2hrs before individual bedtime over the recorded days (%TDEI).
	E2: %TDEI for evening main meal	Continuous data	Mean EI for the biggest EO between 17:00-23:59 over the recorded days (%TDEI).
	E3: %TDEI for evening snack	Continuous data	Mean EI from EOs after the biggest EO between 17:00-23:59 over the recorded days (%TDEI).
EI of EO-categorical variables (NE *)	E1c1: >=30% of TDEI after 18:00	Categorical data	Number of recorded days consuming over 30% of TDEI for 18:00-23:59 (0-3 days for week/weekend; 0-2 days for weekdays; 0-1 days for weekend days)
	E1c2: >=25% of TDEI within 2hrs before bedtime	Categorical data	Number of recorded days consuming over 25% of TDEI within 2hrs before individual bedtime (0-3 days for week/weekend; 0-2 days for weekdays; 0-1 days for weekend days)
Meal frequency	F1: Eating frequency after 17:00	Count data	Median number of EOs between 17:00-23:59 over three recorded days
	F2: Researcher-defined dinner	Categorical data	Number of recorded days having EOs between 17:00-18:29 (0-3 days for week/weekend; 0-2 days for weekdays; 0-1 days for weekend days)
	F3: Researcher-defined evening snack	Categorical data	Number of recorded days having EOs after the biggest EO between 17:00-23:59 (0-3 days for week/weekend; 0-2 days for weekdays; 0-1 days for weekend days)

Abbreviations: EO, eating occasion; EI, energy intake; TDEI, total daily energy intake; NE, night eating. T1: Eating at a late time at night (i.e., a. individual usual bedtime; b. average bedtime). E1: EI from a time frame in the evening/night (i.e., a. specific clock time; b. within 2 hours before bedtime; c. categorical variable derived from continuous variables in E1 to define *NE, including: c1-NE1 and c2-NE2). NE: Night eating was initially defined as eating more after a late time by previous studies, our study adapts the criteria for UK children in ALSPAC.

In terms of EI, on average, children consumed 35.8% of TDEI in the evening (after 17:00), with 27.4% of TDEI from the evening meal, and 5.5% from evening snacks. Notably, there was strong evidence that EI for the evening main meal and evening snack were greater for the week than the weekend. Additionally, more than half of children met the NE criteria on at least one recorded day, with 58.2% consuming more than 25% of TDEI within 2 hours of bedtime (NE1) and 61% consuming more than 30% of TDEI after 18:00 (NE2). However, substantially fewer children were recurrent night eaters, which were classified as maintaining NE for at least two recorded days, with prevalences of 29.2% and 26.2%, respectively, for NE1 and NE2. Sex differences were found in EI for evening snacks only, with greater EI consumed by males than females. No substantial sex differences were found in other EI-related LER variables.

The median count of daily EOs in the evening over three recorded days was 2. More than half of children consumed dinner regularly, with 59.9% consuming dinner on every recorded day. Consuming evening snacks tended to be prevalent, with 65.8% consuming evening snacks for at least two recorded days. The frequency was greater on weekdays than on weekends, for both dinner and evening snacks. Sex differences were found in the consumption of evening snacks only, which was more common in males than females.

### Correlations between LER variables

**Figure 5.**
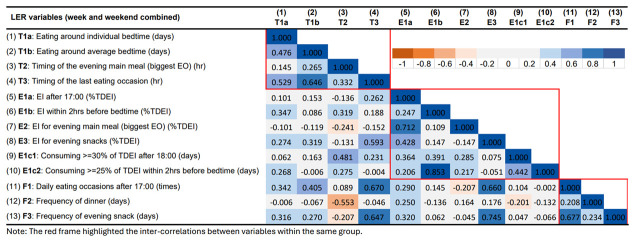
Correlations between variables describing later eating rhythm in the whole week in ALSPAC.

Figure 5 presents the correlations between the LER variables for week/weekend. In general, most correlations were positive. The red frame highlights the correlations between variables within the same domain. Most variables were positively correlated with other variables in the same group. The variables in the timing group were all positively correlated with each other. The timing of the last EO had a strong positive correlation with eating around bedtime. It was also positively correlated with the consumption of evening snacks (both EI and frequency). Very few negative correlations were observed; most negative ones involved the time of the evening main meal (the biggest EO in the evening) which was the only variable that had an equivalent number of positive and negative correlations with other variables. The time of evening main meal was negatively correlated with the frequency of having EOs between 17:00 and 18:29 (usual dinner period), and EI in the evening. In terms of EI, EI in the evening (E1a) were positively correlated with all variables in EI and meal frequency groups, but not timing, and the correlations with EI for evening main meal/evening snacks were stronger. Conversely, EI within 2 hrs before bedtime was mainly correlated with timing-related variables. In terms of meal frequency, more frequent EOs in the evening was strongly correlated with eating at a later time, such as eating around bedtime, later last EO, and more evening snacks (EI for evening snacks and frequency of evening snacks).

**Figure 6.**
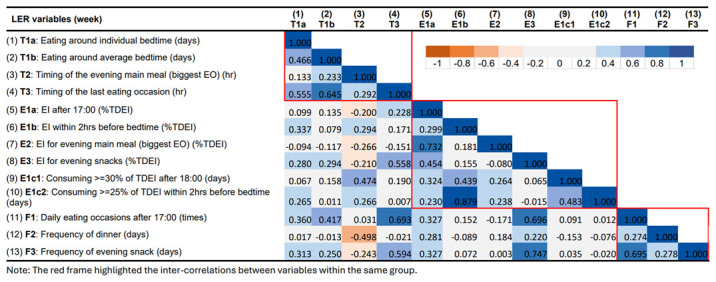
Correlations between variables describing later eating rhythm on weekdays in ALSPAC.

**Figure 7.**
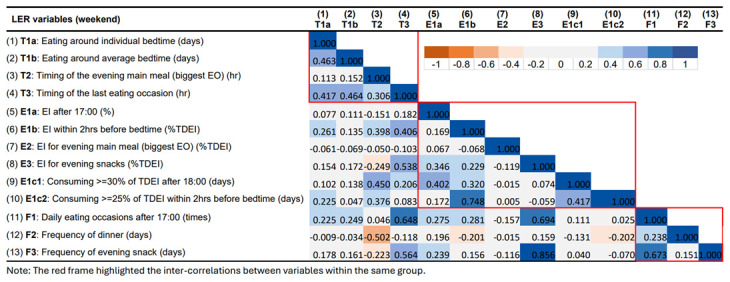
Correlations between variables describing later eating rhythm on weekend days in ALSPAC.

The correlation matrix for LER variables for the week and the weekend are presented in Figure 6 and Figure 7, respectively. Overall, the inter-correlations between the 13 LER variables did not differ substantially between weekday and weekend. Despite the slight differences in the strength of the correlations, they always pointed in the consistent direction.

In summary, most LER variables were positively correlated with each other within the same main group (timing, EI, and meal frequency). Stronger and more consistent inter-correlations were observed in the groups of timing and meal frequency. On the other hand, some of the variables showed strong correlations across groups. Stronger positive associations were found between timing of last EO (T3), EI/frequency for evening snack (E3, F3), and meal frequency in the evening (F1). The time of the evening main meal was involved in most of the negative associations, especially with LER variables in the EI group. For example, a later time of the evening main meal was found to be associated with smaller EI for evening main meal and a lower frequency of evening snacks.

## Strengths and limitations of the data

There are a number of strengths including the use of 3-day food diaries for collecting dietary data, ensuring accurate and detailed recording of food and beverage consumption. Notably, as emphasized in the current 2020 Dietary Guidelines Advisory Committee Report ^43^, to capture habitual/customary eating behaviours, it is vital to collect dietary data for multiple days. Dietary records, also known as food diaries or food records, are often regarded as the “gold standard” against which other dietary methods can be assessed ^44, 45^. Participants are asked to prospectively record the exact time of foods and beverages, and the amounts of each consumed over one or multiple days. In addition, it enabled the type of day (week; weekend) to be recorded, which is important information as eating habits are very likely to be different between the week and weekend ^46– 48^. Since food records are completed while foods are consumed, information on food consumption could be captured more accurately and less likely to be omitted than other methods that are not. However, according to the recent systematic review investigating the association between LER and adiposity in children, very few studies (5/47) used food diaries for at least three days ^28^, due to relatively large respondent burden and high personnel costs ^49^. Despite this, a large number of participants were included in the current study. To address the inconsistent definition of LER used in previous literature, this study applied a large set of comparable and operative definitions of LER, which increases compatibility with other studies. The generation of LER variables and the exploration of the correlations between LER variables can contribute to the understanding of chrono-nutrition, disordered eating, and other time-related eating behaviours in the UK. The data collected and the new variables derived here can be used for further research in these areas and may have implications for interventions aimed at preventing diet-related childhood health.

Key limitations of the study include the use of a subsample of children who had completed food diaries, however those coded for exact times were randomly selected and no biases were introduced. The definitions of LER are contingent upon the availability of dietary and sleep-related data. In addition, bedtime was self-reported by parents as the *usual* bedtime and was not recorded on the same days as the food diaries. This may lead to two problems: 1) the observed bedtime-related variables, such as eating around individual bedtime and eating within 2 hours before bedtime, may not be as accurate as their names implied, and 2) we assumed that children were unlikely to get up and eat after their bedtime, however the reported usual bedtime was earlier than the time of last EO in a substantial group of children (23.7%). Another limitation is that the prevalence of dinner skipping may be overestimated due to the inaccuracy of usual dinner time (17:00–18:29). Children who consumed dinner after 18:29 were therefore counted as dinner skippers. However, this usual dinner time was determined based on the peaks of the distribution of EI during the evening (see Figure 2), thus it is believed that this would be the most appropriate way, similar to previous studies ^50^, to approximate “dinner skipping” when it was unlikely to capture the participants’ perception of dinner using food diaries.

The food diaries used in ALSPAC have shown validity when compared to the UK’s National Diet and Nutrition Survey (NDNS); when comparing intakes from the NDNS using 7–10 year old children in 1997, very similar results were reported ^51^. The same methodology has also been used at younger ages and dietary intakes have correlated highly with relevant blood markers ^52^. 

To enhance the accuracy of measuring meal-related variable and comparability with other dietary measures, we would recommend that future studies using food diaries include bedtime and participant-identified meal types for each EO. Specifically, food diaries could be improved by asking participants to label each eating occasion (EO) with a self-defined meal type (e.g., breakfast, lunch, dinner or snack).

## Ethical approval and Consent

Ethical approval for the study was obtained from the ALSPAC Ethics and Law Committee and the Local Research Ethics Committees. Informed consent for the use of data collected via questionnaires and clinics was obtained from participants following the recommendations of the ALSPAC Ethics and Law Committee at the time. Study participants have the right to withdraw their consent for elements of the study or from the study entirely at any time. Full details of the ALSPAC consent procedures are available on the study website ( http://www.bristol.ac.uk/alspac/researchers/research-ethics/).

## Data Availability

ALSPAC data access is through a system of managed open access. The steps below highlight how to apply for access to the data included in this data note and all other ALSPAC data: 1. Please read the ALSPAC access policy ( www.bristol.ac.uk/media-library/sites/alspac/documents/researchers/data-access/ALSPAC_Access_Policy.pdf) which describes the process of accessing the data and samples in detail, and outlines the costs associated with doing so. 2. You may also find it useful to browse our fully searchable research proposals database ( https://proposals.epi.bristol.ac.uk/?q=proposalSummaries), which lists all research projects that have been approved since April 2011. 3. Please submit your research proposal ( https://proposals.epi.bristol.ac.uk/) for consideration by the ALSPAC Executive Committee. You will receive a response within 10 working days to advise you whether your proposal has been approved.
