# Supplementary material for: Later eating rhythm measured in children at 7 years of age in the ALSPAC cohort
Source: Wellcome Open Res. 2024 Aug 21;9:77. Originally published 2024 Feb 19. [Version 3] doi: 10.12688/wellcomeopenres.20605.3 (PMC11393536; doi:10.12688/wellcomeopenres.20605.3)
Supplement: Peer review history

# Reviewer report: Reviewer response for version 3

**DOI:** 10.21956/wellcomeopenres.25322.r98700

**Published:** 2024-10-04

**Authors:** Emily N. C Manoogian

**Affiliations:** 1 Salk Institute for Biological Studies, La Jolla, California, USA

**Summary:**

 The authors assessed eating time in a large cohort of 7-year-old children from the ALSPAC cohort. Participants logged the timing of all energy intake and medications. This is a large data set, and although it is not perfect (more days per person would make it much stronger), it is much stronger than anything else available for this age. This is a strong database to share with the community.

**Minor Issues**

**Abstract:**

 It would be helpful to have a concluding sentence similar to the end of the introduction about how you expect the data to be used.

**Methods Questions:**

 Did you control for a minimum number of meals in a day? For instance, if someone only logged one item in the evening, was that excluded as a non-compliant logging day or was it taken as fact? In figure 3, There multiple entries with 100% of TEI happening after 4, 5, 6, or event 7pm. This seems more like incomplete entries rather than reality. Please explain how you handled this issue.

 What do you mean 1,834 were selected based on criteria and 3,035 participants were selected randomly? Isn’t the 4,869 the total number of participants that had exact food timing? This would mean you used all available data with exact time, rather than selecting them. Please clarify.

 Did you assess if late night eating was associated with medication? Is it possible children needed to take a medication with food that was outside there normal patterns?

**Figures/Tables:**

 Figure 4 needs more labels on the axis and a better description in the legend.

**Table 5.** Add definition in table for LER variabiles, specifically T1a and T1b, ie how you define eating around individual bedtime. I realize it is available elsewhere in the text, but it makes it much easier on the reader to have it in the table, and there is room.

Are sufficient details of methods and materials provided to allow replication by others?

Partly

Is the rationale for creating the dataset(s) clearly described?

Yes

Are the datasets clearly presented in a useable and accessible format?

Yes

Are the protocols appropriate and is the work technically sound?

Yes

Reviewer Expertise:

Chronobiology with a specialty in temporal eating patterns in humans.

I confirm that I have read this submission and believe that I have an appropriate level of expertise to confirm that it is of an acceptable scientific standard.

# Reviewer report: Reviewer response for version 3

**DOI:** 10.21956/wellcomeopenres.25322.r94418

**Published:** 2024-09-12

**Authors:** Henna Vepsäläinen

**Affiliations:** 1 Department of Food and Nutrition, University of Helsinki, Helsinki, Finland

The authors have sufficiently addressed the issues raised.

Are sufficient details of methods and materials provided to allow replication by others?

Partly

Is the rationale for creating the dataset(s) clearly described?

Yes

Are the datasets clearly presented in a useable and accessible format?

Partly

Are the protocols appropriate and is the work technically sound?

Yes

Reviewer Expertise:

Nutritional epidemiology, public health nutrition, nutrition and wellbeing in families

I confirm that I have read this submission and believe that I have an appropriate level of expertise to confirm that it is of an acceptable scientific standard.

# Reviewer report: Reviewer response for version 3

**DOI:** 10.21956/wellcomeopenres.25322.r94419

**Published:** 2024-09-02

**Authors:** Yangbo Sun

**Affiliations:** 1 Preventive Medicine, The University of Tennessee Health Science Center, Memphis, Tennessee, USA

The authors have addressed all the comments. I have no further comment.

Are sufficient details of methods and materials provided to allow replication by others?

Yes

Is the rationale for creating the dataset(s) clearly described?

Partly

Are the datasets clearly presented in a useable and accessible format?

Yes

Are the protocols appropriate and is the work technically sound?

Yes

Reviewer Expertise:

nutritional epidemiology

I confirm that I have read this submission and believe that I have an appropriate level of expertise to confirm that it is of an acceptable scientific standard.

# Reviewer report: Reviewer response for version 2

**DOI:** 10.21956/wellcomeopenres.25185.r91323

**Published:** 2024-08-15

**Authors:** Yangbo Sun

**Affiliations:** 1 Preventive Medicine, The University of Tennessee Health Science Center, Memphis, Tennessee, USA

Thanks for providing the revised manuscript. It is much approved and address most of the questions. One minor comment: it is still unclear about the description/comment/discussion on the reliability and validation on the measures of food dairies.

Are sufficient details of methods and materials provided to allow replication by others?

Yes

Is the rationale for creating the dataset(s) clearly described?

Partly

Are the datasets clearly presented in a useable and accessible format?

Yes

Are the protocols appropriate and is the work technically sound?

Yes

Reviewer Expertise:

nutritional epidemiology

I confirm that I have read this submission and believe that I have an appropriate level of expertise to confirm that it is of an acceptable scientific standard, however I have significant reservations, as outlined above.

# Response

**Published:** 2024-08-16

**Authors:** Kate Northstone

**Affiliations:** University of Bristol, UK

We respond to second reviewer's comments as follows:  

1. Thanks for providing the revised manuscript. It is much approved and address most of the questions. One minor comment: it is still unclear about the description/comment/discussion on the reliability and validation on the measures of food dairies.
**Response:** *Thank you for your continued feedback. We appreciate your insights and have taken additional steps to clarify the discussion on the reliability and validation of food diaries - more generally but also those used specifically in ALSPAC in the penultimate paragraph of the Strengths and limitations section (it is not clear which in particular you might be referring to and so hope we have attended to both sufficiently).* *We hope these additions address your concerns, and we thank you again for your valuable input.*

# Reviewer report: Reviewer response for version 2

**DOI:** 10.21956/wellcomeopenres.25185.r91324

**Published:** 2024-08-13

**Authors:** Henna Vepsäläinen

**Affiliations:** 1 Department of Food and Nutrition, University of Helsinki, Helsinki, Finland

Thank you for providing an amended version of the data note. This is an important contribution to the scientific community and research groups investigating eating frequency and meal patterns in children. A few minor comments to the authors can be found below.

1. It is still unclear to me how the subsample of 4,869 food diaries were selected. Apparently some other data was used as the inclusion criteria for 1,834 diaries, which data were these? Why? Was the number of diaries chosen randomly steered by power calculations? More details about the selection of the subsample would be appreciated.
2. Table 3: I am a bit confused with Table 3, which is referred to define eating occasions but also LER variables. Should the latter refer to Table 4?

Are sufficient details of methods and materials provided to allow replication by others?

Partly

Is the rationale for creating the dataset(s) clearly described?

Yes

Are the datasets clearly presented in a useable and accessible format?

Partly

Are the protocols appropriate and is the work technically sound?

Yes

Reviewer Expertise:

Nutritional epidemiology, public health nutrition, nutrition and wellbeing in families

I confirm that I have read this submission and believe that I have an appropriate level of expertise to confirm that it is of an acceptable scientific standard, however I have significant reservations, as outlined above.

# Response

**Published:** 2024-08-16

**Authors:** Kate Northstone

**Affiliations:** University of Bristol, UK

We respond to first reviewers comments as follows:

1. It is still unclear to me how the subsample of 4,869 food diaries were selected. Apparently some other data was used as the inclusion criteria for 1,834 diaries, which data were these? Why? Was the number of diaries chosen randomly steered by power calculations? More details about the selection of the subsample would be appreciated.
**Response: **T *hank you for your comments and apologies for not explaining this sufficiently.* *To clarify, the 1,834 food diaries were selected based on the completion of the Adult Eating Behaviour Questionnaire (AEBQ), which was part of a self-completion questionnaire sent to participants when they were 24 years old. This selection was made based on a previous project conducted around 2017 that initiated the coding of exact times in participants that also had eating behaviour at 24 years measured, as this was the outcome of interest. We have added clarification to the next  version of this paper. After this initial selection, we continued coding the remaining food diaries which were randomly selected. This resulted in a total of 4,869 out of 7,285 diaries coded with precise timing of food intake up to date.* *The selection of these diaries was not guided by power calculations. The initial batch of coding was for another PhD project. Our goal was to top these up randomly in order to minimize selection bias. We carefully examined potential differences between the children included (n=4,869) and those excluded from the study. As shown in Table 2, there were no real differences between these groups, indicating that the included subsample is representative of the entire cohort, and no biases were introduced.*  
2. Table 3: I am a bit confused with Table 3, which is referred to define eating occasions but also LER variables. Should the latter refer to Table 4?
**Response:** *We appreciate your careful review. We have corrected the table numbers in the text, so the information about LER variables now correctly refers to Table 4 instead of Table 3.*

# Reviewer report: Reviewer response for version 1

**DOI:** 10.21956/wellcomeopenres.22808.r75404

**Published:** 2024-05-08

**Authors:** Yangbo Sun

**Affiliations:** 1 Preventive Medicine, The University of Tennessee Health Science Center, Memphis, Tennessee, USA

Thanks for the opportunity to review this interesting paper. The authors used ALSPAC dataset to describe the late eating patterns among children. I have a few suggestions for the authors to consider.

1. It is unclear about the validity of the food diaries collection in the study
2. It will be more informative if the authors can compare the children included in this study and those excluded
3. the authors did not compare their findings with other studies, which will provide more information

Are sufficient details of methods and materials provided to allow replication by others?

Yes

Is the rationale for creating the dataset(s) clearly described?

Partly

Are the datasets clearly presented in a useable and accessible format?

Yes

Are the protocols appropriate and is the work technically sound?

Yes

Reviewer Expertise:

nutritional epidemiology

I confirm that I have read this submission and believe that I have an appropriate level of expertise to confirm that it is of an acceptable scientific standard, however I have significant reservations, as outlined above.

# Response

**Published:** 2024-07-22

**Authors:** Kate Northstone

**Affiliations:** University of Bristol, UK

We thank the reviewers for their positive and constructive comments, and we hope that our revisions addressed their queries and suggestions. We present the reviewers comments below, and provide our responses in italics.

**Reviewer 1:**

1. Figure 1 + related text: Figure includes information that is not explained in the text – what is the justification for selecting only a share of the food diaries for investigating the timing of consumption? I understand that the procedure was burdensome and this would be a perfectly acceptable reason for not including all diaries, but maybe you performed a sample size calculation to estimate how many diaries would be needed to explore specific research questions and used that as a justification for including ~4000 diaries or something like that? In addition, it is somewhat unclear what some of the Ns in the figure represent (for example N=4029; 4063; 3757) since the text explaining this comes later. Maybe the figure could be moved later?
2. Later along in the text, it is mentioned that “Among these, 1,834 were selected based on the availability of other data, while 3,035 were selected randomly.” Based on this, I would remove the word “randomly” from Figure 1, as this was not the case for all of the 4869 diaries.
**Response:** *Thank you for these comments regarding Figure 1. To clarify the information, we have moved this figure to now appear after the explanatory text (where Figure 1 is clearly referenced). As the LER variables require precise timing of food intake, this data note focuses on those diaries which had precise time entries. We will update this data note in the future when the remaining diaries are entered. To address your comments on randomisation, we have removed the word “randomly” from Figure 1. Additionally, we have created a new table (Table 2) showing the characteristics of children included in this study and those excluded due to not have precise timings, to provide a clearer understanding of the sample selection.*
3. Table 2: There probably is no universal definition for a breakfast (is it a main meal or more like a snack), but using the definition in Table 2, is it possible that for example breakfast and lunch are within the same time slot (between 6:00 and 11:59), or lunch and dinner (between 12:00 and 16:59)? This would then result in some of the “main meals” to be coded as snacks – how would that effect the results and is there any solution that would prevent this from happening?
**Response:** *Thank you for this question. It is indeed challenging to determine the self-defined meal types from a food diary, as noted in the limitations section of this paper. This limitation may result in some main meals being coded as snacks, particularly if breakfast and lunch, or lunch and dinner, fall within the same time slot (e.g., between 06:00 and 11:59 or 12:00 and 16:59).* *However, as this paper focuses on eating later in the day, the potential misclassification of main meals as snacks does not significantly impact the core findings related to later eating behaviour. We have defined the time frames based on usual meal times, as shown in Figure 2, to minimize bias and improve the accuracy of our assessments. Despite these efforts, we acknowledge that the inability to capture participants' perceptions of what constitutes a dinner or evening snack remains a limitation. Note that Table 2 referred to by the reviewer is now Table 3.* T *o address this issue in future studies, it may be beneficial to ask participants to label each eating occasion with a self-defined meal type, as this could provide a more accurate estimation of meal-related variables. We have added this recommendation for future dietary assessment to the Strengths and Limitations section.*
4. Table 3: This table is nicely compiled. However, some of the definitions are a bit unclear, for example T1a: is the final variable a continuous or categorical variable, if it is categorical, which are the categories? From the abstract it can be deducted that this is a binary variable, maybe a column indicating the type of the variable could be added?
**Response:** *We have now added a column indicating the type of variable in Table 3.*  
5. Night eating (NE) is mentioned in the text and Table 3, but not in the abstract. For the sake of clarity, the variable names should remain the same throughout the text, although I am not sure whether night eating is an appropriate term when the participants are children and the authors have also touched upon this topic in the introduction. Later also recurrent night eating is mentioned – should this be included in the definitions of Table 3?
**Response:** *We have added “NE” next to its definition in the abstract for clarity. NE is a categorical variable indicating how many days out of the recorded days that children exhibit NE behaviour. The term “Recurrent NE” is used in the text to describe children who exhibit NE behaviour on all three recorded days, which is an extension of the existing definition of NE in Table 3. We have clarified this in the text with brackets but will not add it to Table 3, as that simply provides a description of the frequency of NE.*
6. Table 4: Even though the variables have been explained in Table 3, it should also readable on its own, and thus, I recommend that the variables are briefly defined in this table as well (for example, what does “Eating around individual bedtime” mean).
**Response:** *Thank you for this feedback. Considering the complexity and size of Table 4, we believe that duplicating the definitions would make it more cumbersome. Table 3 already provides clear definitions, and we hope that readers can easily refer to it for detailed explanations. To facilitate this, we have now included a note in Table 4 directing readers to Table 3 for variable definitions. We hope that this approach will maintain clarity without complicating Table 4 further.*
7. Tables 5, 6 and 7: I am not sure if the color coding (and the red frame referred to in the footnote) is working as designed, for me it seems like there is normal table with correlation coefficients.
**Response:** *We believe this is down to formatting issues on the website version. They have now been converted to figures so hopefully the colour coding is maintained compared to the original.*
8. Referring to question #3, would you recommend researchers to use participant-defined meals or to categorize meals according to time slots? Why? Some kinds of recommendations could be incorporated into the Strengths and limitations of the data to steer future research.
**Response:** *Thank you for this suggestion. Please refer to our response to Comment 3.*
9. The authors state that, due to the randomization, it is unlikely that bias was introduced. This could be easily checked by comparing those included (randomized into the current study) and excluded, if appropriate data is available (for example sex, parental education or income etc.).
**Response:** *Thank you for your suggestion. Please refer to our response to Comment 2, where we have addressed this by adding a table in the appendix that displays the characteristics of children included and excluded from the study side by side. This comparison should provide the necessary information to assess any potential bias due to randomization.*
10. It is mentioned that dietary records are regarded as the “gold standard” and that to capture habitual/customary eating behaviours, it is vital to collect dietary data for multiple days. For some food groups that are no that often consumed, such as fish, three days may not be enough, and some other dietary assessment methods may be better in assessing those. However, this study focused solely on the timing of eating. It would have been nice to read how well the authors think a 3-day diary is able to capture the timing of eating? Is three days enough and if so, why? How many days you recommend should be recorded? **Response:** *Thank you for your feedback. As mentioned in the Strengths and Limitations section, it is emphasized in the current 2020 Dietary Guidelines Advisory Committee Report, to capture a habitual/customary eating behaviour, it is necessary to use at least three days of data collection. While three days may not be sufficient for accurately capturing the intake of rarely consumed food groups, such as fish, this study focused solely on the timing of eating. We believe that three days are sufficient to capture the typical timing of eating occasions. However, for studies focusing on specific food groups with less frequent consumption, a longer recording period might be necessary.* *Please also note that the lead author’s affiliation has changed given a change of job.*

**Reviewer 2:**

1. It is unclear about the validity of the food diaries collection in the study
**Response:** *Thank you for your comment. We have provided evidence of the validity of food diaries at the end of the Strengths and Limitations section of the Discussion.*
2. It will be more informative if the authors can compare the children included in this study and those excluded
  ​​​​​​​ **Response:** *We have added a new table (Table 2) that displays the characteristics of children included in the study compared to those not included due to not having precise timings. This comparison should provide more informative insights into the differences between the two groups.*
3. The authors did not compare their findings with other studies, which will provide more information.
**Response:** *Thank you for this comment. This paper is a data note and as such focusses on describing how later eating rhythm (LER) variables have been derived from ALSPAC dietary data for the first time. The primary aims are to introduce the dataset and encourage future research studies. We have highlighted the strengths of this dataset compared to others in the field within the Discussion section. However, since this is not a research paper, making direct comparisons with findings from other studies falls outside the scope of this work.*

# Reviewer report: Reviewer response for version 1

**DOI:** 10.21956/wellcomeopenres.22808.r76725

**Published:** 2024-04-17

**Authors:** Henna Vepsäläinen

**Affiliations:** 1 Department of Food and Nutrition, University of Helsinki, Helsinki, Finland

Thank you for the opportunity to review this carefully written data note, which describes the formation of variables used to assess later eating rhythm among children. However, I hope that the authors will be willing to clarify some parts of the paper to make it easier to follow, which will in turn help other researchers in using the same types of variables, where applicable. I also wish for a few sharp recommendations for future research. Please find more detailed questions and comments below.

1. Figure 1 + related text: Figure includes information that is not explained in the text – what is the justification for selecting only a share of the food diaries for investigating the timing of consumption? I understand that the procedure was burdensome and this would be a perfectly acceptable reason for not including all diaries, but maybe you performed a sample size calculation to estimate how many diaries would be needed to explore specific research questions and used that as a justification for including ~4000 diaries or something like that? In addition, it is somewhat unclear what some of the Ns in the figure represent (for example N=4029; 4063; 3757) since the text explaining this comes later. Maybe the figure could be moved later?
2. Later along in the text, it is mentioned that “Among these, 1,834 were selected based on the availability of other data, while 3,035 were selected randomly.” Based on this, I would remove the word “randomly” from Figure 1, as this was not the case for all of the 4869 diaries.
3. Table 2: There probably is no universal definition for a breakfast (is it a main meal or more like a snack), but using the definition in Table 2, is it possible that for example breakfast and lunch are within the same time slot (between 6:00 and 11:59), or lunch and dinner (between 12:00 and 16:59)? This would then result in some of the “main meals” to be coded as snacks – how would that effect the results and is there any solution that would prevent this from happening?
4. Table 3: This table is nicely compiled. However, some of the definitions are a bit unclear, for example T1a: is the final variable a continuous or categorical variable, if it is categorical, which are the categories? From the abstract it can be deducted that this is a binary variable, maybe a column indicating the type of the variable could be added?
5. Night eating (NE) is mentioned in the text and Table 3, but not in the abstract. For the sake of clarity, the variable names should remain the same throughout the text, although I am not sure whether night eating is an appropriate term when the participants are children and the authors have also touched upon this topic in the introduction. Later also recurrent night eating is mentioned – should this be included in the definitions of Table 3?
6. Table 4: Even though the variables have been explained in Table 3, it should also readable on its own, and thus, I recommend that the variables are briefly defined in this table as well (for example, what does “Eating around individual bedtime” mean).
7. Tables 5, 6 and 7: I am not sure if the color coding (and the red frame referred to in the footnote) is working as designed, for me it seems like there is normal table with correlation coefficients.
8. Referring to question #3, would you recommend researchers to use participant-defined meals or to categorize meals according to time slots? Why? Some kinds of recommendations could be incorporated into the Strengths and limitations of the data to steer future research.
9. The authors state that, due to the randomization, it is unlikely that bias was introduced. This could be easily checked by comparing those included (randomized into the current study) and excluded, if appropriate data is available (for example sex, parental education or income etc.).
10. It is mentioned that dietary records are regarded as the “gold standard” and that to capture habitual/customary eating behaviours, it is vital to collect dietary data for multiple days. For some food groups that are no that often consumed, such as fish, three days may not be enough, and some other dietary assessment methods may be better in assessing those. However, this study focused solely on the timing of eating. It would have been nice to read how well the authors think a 3-day diary is able to capture the timing of eating? Is three days enough and if so, why? How many days you recommend should be recorded?

Are sufficient details of methods and materials provided to allow replication by others?

Partly

Is the rationale for creating the dataset(s) clearly described?

Yes

Are the datasets clearly presented in a useable and accessible format?

Partly

Are the protocols appropriate and is the work technically sound?

Yes

Reviewer Expertise:

Nutritional epidemiology, public health nutrition, nutrition and wellbeing in families

I confirm that I have read this submission and believe that I have an appropriate level of expertise to state that I do not consider it to be of an acceptable scientific standard, for reasons outlined above.
